# Preclinical Evaluation of Vemurafenib as Therapy for BRAF^V600E^ Mutated Sarcomas

**DOI:** 10.3390/ijms19040969

**Published:** 2018-03-23

**Authors:** Sarina Gouravan, Leonardo A. Meza-Zepeda, Ola Myklebost, Eva W. Stratford, Else Munthe

**Affiliations:** 1 Department of Tumor Biology, Institute for Cancer Research, Oslo University Hospital, 0379 Oslo, Norway; sarina.gouravan@rr-research.no (S.G.); Leonardo.A.Meza-Zepeda@rr-research.no (L.A.M.-Z.); ola.myklebost@uib.no (O.M.); 2 Genomics Core Facility, Department of Core Facility, Institute for Cancer Research, Oslo University Hospital, 0379 Oslo, Norway; 3 Department of Clinical Science, University of Bergen, Bergen, Norway

**Keywords:** BRAF, precision medicine, drug repurposing

## Abstract

The BRAF^V600E^ mutation, which in melanoma is targetable with vemurafenib, is also found in sarcomas and we here evaluate the therapeutic potential in sarcoma cell lines. **Methods**: Four sarcoma cell lines harboring the BRAF^V600E^ mutation, representing liposarcomas (SA-4 and SW872), Ewing sarcoma (A673) and atypical synovial sarcoma (SW982), were treated with vemurafenib and the effects on cell growth, apoptosis, cell cycle progression and cell signaling were determined. **Results**: Vemurafenib induced a strong cytostatic effect in SA-4 cells, mainly due to cell cycle arrest, whereas only moderate levels of apoptosis were observed. However, a high dose was required compared to BRAF^V600E^ mutated melanoma cells, and removal of vemurafenib demonstrated that the continuous presence of drug was required for sustained growth inhibition. A limited growth inhibition was observed in the other three cell lines. Protein analyses demonstrated reduced phosphorylation of ERK during treatment with vemurafenib in all the four sarcoma cell lines confirming that the MAPK pathway is active in these cell lines, and that the pathway can be inhibited by vemurafenib, but also that these cells can proliferate despite this. **Conclusions**: These findings indicate that vemurafenib alone would not be an efficient therapy against BRAF^V600E^ mutated sarcomas. However, further investigations of combination with other drugs are warranted.

## 1. Introduction

Sarcoma is a heterogeneous group of rare tumors, generally arising from mesenchymal cells and accounting for approximately 1% of all human cancers and 10–20% of childhood solid cancers [1,2]. Despite the rareness of the disease, the high degree of mortality in children and adolescents with sarcomas accounts for many years of lost lives [2]. More than 50 subtypes of sarcoma have been classified by the World Health Organization [3], and these can be divided into two main subgroups; bone and soft tissue tumors. With the exception of imatinib repurposed for patients with gastrointestinal stromal tumor (GIST) [4], targeted therapies are not routinely used for sarcomas, and the main treatments are still surgery, chemotherapy and radiotherapy, sometimes in combination. Unfortunately, in many cases these treatments are not curative, and improved treatment strategies are much needed. Rare cancers, such as these, are generally not a focus of drug development, and therefore repurposing drugs already approved for other indications would be a valuable alternative. Inhibitors targeting cancer-specific mutations are ideal for repurposing. The presence of the exact same mutation in other cancer types suggests that the cancer may be driven by the same mechanism. However, different context requires validation of the therapeutic principle since other compensatory factors may enable cells with different phenotypes to escape therapy.

Mutated BRAF are described in several cancers and can be targeted by selective inhibitors, such as vemurafenib [5]. The RAF kinase family (ARAF, BRAF and RAF1) are serine/threonine kinases which signal through the mitogen-activated protein kinase (MAPK) pathway, including the down-stream kinases MEK and ERK [6]. The cascade is initiated upon binding of a mitogen or a growth factor, such as PDGF and EGF, to its receptor tyrosine kinase (RTK) in the cell membrane. This leads to a RAS-induced phosphorylation of RAF proteins and initiation of a MAPK phosphorylation cascade resulting in activation of transcription factors that regulate cell proliferation and/or apoptosis [7,8]. Several mutations in the BRAF gene have been described, but the most common genetic alteration results in a substitution of the amino acid 600 from valine to glutamic acid (BRAF^V600E^) [9]. This amino acid is within the activation loop, and the substitution renders the protein constitutively active and independent of RAS activation [10,11].

The BRAF^V600E^ mutation accounts for more than 90% of all reported BRAF mutations [12]. The BRAF^V600E^ mutation is present in 50% of melanomas and has also been identified in subsets of many other cancer types, including colorectal cancer (10–15%), papillary thyroid carcinoma (30–50%) and non-small cell lung cancer (1.3%) [13,14,15,16,17]. The BRAF^V600E^ mutation has also been reported in GIST, several cases of clear cell sarcoma, and liposarcoma [18,19,20,21]. Furthermore, the BRAF^V600E^ mutation has been described in sarcoma cell lines of different subtypes, including SW872, SA-4, A673 and SW982 [12,21,22].

Vemurafenib was initially approved by the Food and Drug Administration (FDA) in 2011 as a therapy for melanoma patients with mutated BRAF^V600E^, and resulted in a response rate of >50% and approximately 7 months of median progression-free survival for melanoma patients [10,23]. In colorectal patients with the BRAF^V600E^ mutation; however, vemurafenib as monotherapy was unsuccessful due to resistance, which occurred as a consequence of a rapid, EGFR-induced reactivation of the MAPK pathway, observed already at 24–48 h after treatment [14,24]. Importantly, significant tumor regression was achieved when vemurafenib was given in combination with EGFR inhibitors [14]. Vemurafenib has shown some anecdotal therapeutic potential in sarcomas, as a patient with clear cell sarcoma harboring the BRAF^V600E^ mutation was successfully treated with vemurafenib and displayed tumor regression after 8 weeks of treatment [25]. Furthermore, antitumor activity was also observed for a GIST patient treated with dabrafenib, another BRAF^V600E^ inhibitor [20]. Here, we have further investigated the potential of vemurafenib as a therapy for BRAF^V600E^ mutated sarcomas, by determining its efficacy in vitro in four BRAF^V600E^ mutated sarcoma cell lines.

## 2. Results

### 2.1. Expression of BRAF^V600E^ in Sarcoma Cell Lines

We selected four ATCC sarcoma cell lines carrying heterozygous mutation for BRAF^V600E^, namely SA-4 and SW872, classified as liposarcomas, as well as Ewing sarcoma cell line A673 and an atypical synovial sarcoma cell line SW982 lacking the SYT-SSX translocation. The WM9 melanoma cell line, known to harbor a hemizygous BRAF^V600E^ mutation, was included as a positive control, whereas the LPS510 liposarcoma cell line was included as a negative control line for all BRAF^V600E^ mutated sarcoma cell lines, as it is wild-type for BRAF according to our DNA sequencing data. We evaluated the expression of wild-type and mutated BRAF mRNA by next-generation sequencing and found similar levels of wild-type and mutated BRAF transcripts in both SA-4 and SW872 cell lines, whereas LPS510 only expressed wild-type BRAF (Table 1). RNA sequencing data was not available for SW982 and A673 cell lines.

**Figure 1:**
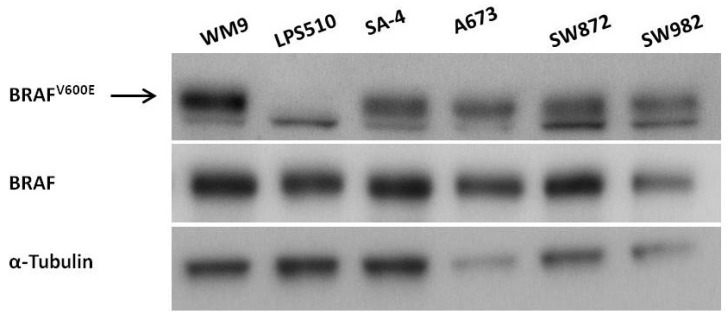
Expression of BRAF^V600E^ protein in sarcoma cell lines. The presence of BRAF^V600E^ mutated protein was evaluated in the five sarcoma cell lines (LPS510, SA-4, A673, SW872, SW982). The melanoma cell line WM9 (with BRAF^V600E^) and LPS510 (with wild-type BRAF) were included as control cell lines. Cell lysates were analyzed by western blotting, using indicated antibodies. α-Tubulin served as protein loading control. Arrow indicates the expected size of BRAF^V600E^, and an unspecific band was observed below this band for all cell types.

To determine the levels of total and BRAF^V600E^ mutated protein in all cell lines, western blotting was performed using antibodies specific for pan-BRAF and BRAF^V600E^, respectively. BRAF protein was abundantly expressed in all the cell lines, and the presence of BRAF^V600E^ mutated protein was confirmed in all five cell lines known to harbor the mutation (Figure 1). The specificity of detection was demonstrated by the absence of mutated protein in the BRAF wild-type cell line, LPS510.

### 2.2. Vemurafenib Inhibits Cell Growth in BRAF Mutated Cell Lines

**Figure 2:**
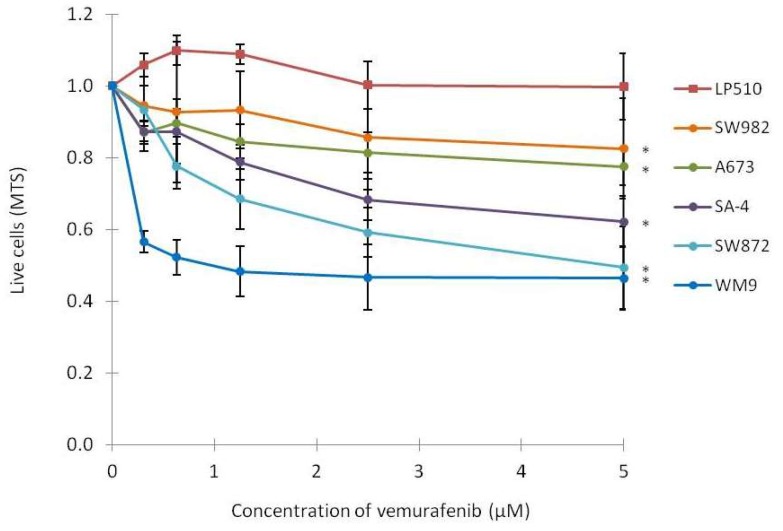
Effect on cell growth following vemurafenib treatment. Cell lines were treated with the indicated concentrations of vemurafenib or vehicle only, and MTS assay was performed 72 h (WM9, LPS510, SA-4 and A673) or 96 h (SW872 and SW982) after initiation of treatment. The absorbance at 450 nm was measured and normalized to vehicle-treated cells. Curves represent means of three biological experiments and error bars represent standard deviations (SD) (*n* = 3). * indicates *p* < 0.05 at the highest dose.

The growth of the cell lines was assessed by MTS assay after 72–96 h of exposure to 0.31 to 5 µM vemurafenib (Figure 2). The SW872 and SA-4 cell lines displayed the most significant reduction in cell growth at all doses, although to a lesser degree than observed for the melanoma cell line WM9. In contrast, A673 and SW982 cell lines displayed a minor response, although significant at the highest dose. As expected, no reduction in cell growth was observed for LPS510 following vemurafenib treatment.

**Figure 3:**
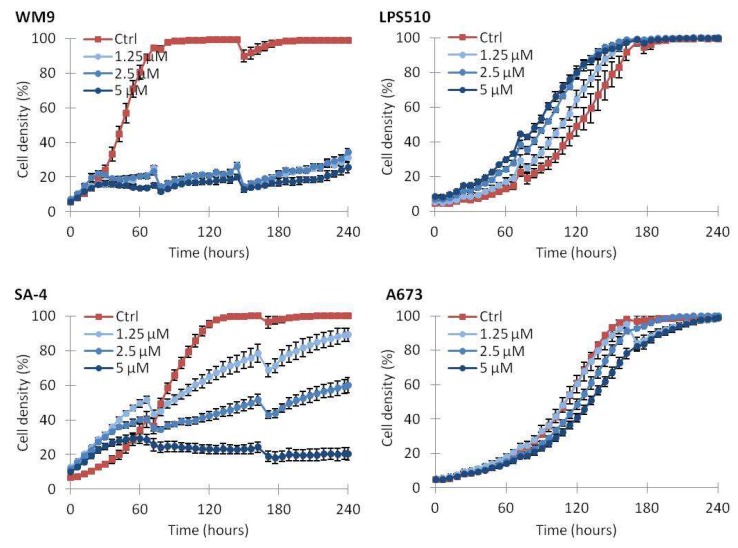

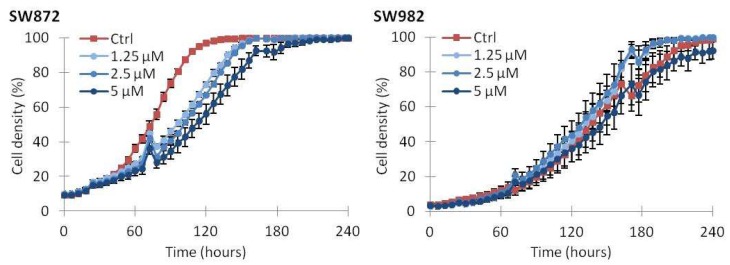
Effect on cell growth during vemurafenib treatment. Cells were treated with the indicated concentrations of vemurafenib or vehicle only (Ctrl) and monitored by time-lapse microscopy. Growth medium supplemented with drug was replaced twice a week. Irregularities in growth curves are caused by loss of poorly attached cells by media change. One representative experiment is shown (*n* = 2). Error bars represent standard error of mean (SEM) of three technical replicates.

Since MTS is a short-term assay with a fixed endpoint that measures cellular metabolic activity, we investigated long term effects of vemurafenib treatment by monitoring cell growth over longer periods of time. Cell cultures were monitored by time-lapse microscopy for up to 240 h in the presence of drug (Figure 3). A rapid and complete growth inhibition was observed for WM9 cells. SA-4 cells also displayed a full growth inhibition, although the inhibition appeared at a later time point and required a higher dose than for WM9 cells. The SW872 cells showed an initial response for the first 72 h, similar to SA-4. However, the inhibition was not sustained as cells exposed to continuous treatment with high doses of vemurafenib reached full cell density approximately 50 h after control-treated cells. The other BRAF^V600E^ mutated sarcoma cell lines showed minor or no responses. The wild-type LPS510 cell line did not display a growth inhibitory response following vemurafenib treatment, but the cells underwent slight changes in morphology, appearing as increased cell density for vemurafenib-treated cells in the time-lapse analysis.

### 2.3. Vemurafenib Induces a Low Level of Apoptosis in SA-4 Cells

**Figure 4:**
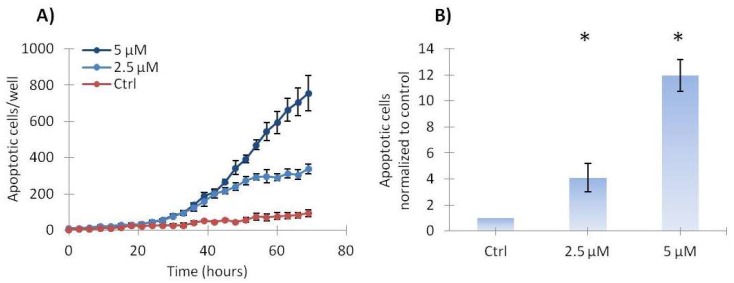
Vemurafenib induces apoptosis in SA-4 cells. (**A**) The number of apoptotic cells per well was determined based on caspase-3/7 activity monitored by time-lapse microscopy during treatment with 2.5 or 5 µM vemurafenib or vehicle only (Ctrl). The curve from one out of three representative experiment is shown and error bars represent standard error of mean (SEM) of three technical replicates; (**B**) Apoptotic cells shown relative to total cells, normalized to control at 72 h. Error bars represent standard deviations (SD) between biological experiments (*n* = 3). * indicates *p* < 0.05.

The best responding cell line, SA-4, was used to investigate in more depth the growth inhibitory mechanisms involved. The effect of 2.5 and 5 µM vemurafenib on apoptosis was evaluated using time-lapse microscopy in the presence of an agent that reports active caspase-3/7. As shown in Figure 4A, a dose-dependent increase in the number of caspase-3/7-positive SA-4 cells was observed, indicating induction of apoptosis. Figure 4B shows the levels of apoptosis relative to control cells after 72 h of treatment, confirming the significant, dose-dependent induction of apoptosis. However, apoptosis was only observed in a low percentage of the cells, suggesting that apoptosis is only a minor contributing factor to the observed growth inhibition.

### 2.4. Vemurafenib Induces G1 Arrest in SA-4 Cells

**Figure 5:**
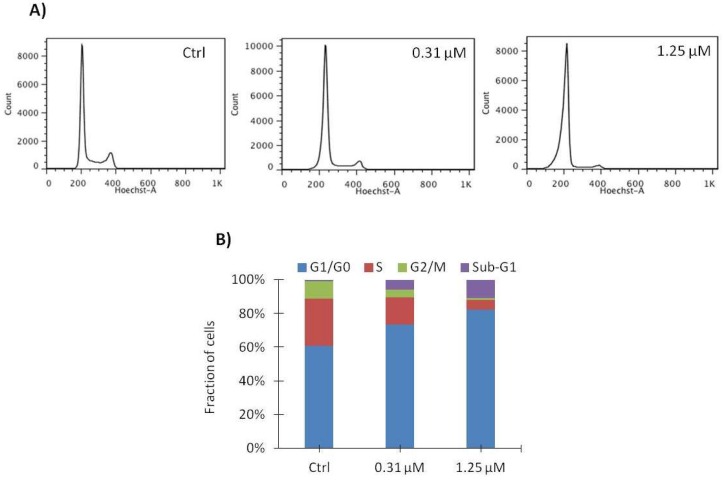
Vemurafenib induces G1 arrest in SA-4 cells. Cells were treated with 0.31 or 1.25 μM vemurafenib or vehicle only (Ctrl) for 48 h and subsequently stained with a DNA-binding dye and analyzed by flow cytometry. (**A**) Histogram from a representative experiment is shown (*n* = 3), where the *x*-axis represents fluorescence intensity and the *y*-axis represents cell count; (**B**) Bar graphs represent distribution of cells among the phases of cell cycle. The fraction of cells in each phase is indicated by colors.

To determine the effect of vemurafenib on cell cycle progression, SA-4 cells were treated with 0.31 or 1.25 µM vemurafenib for 48 h, and subsequently their DNA content was measured by flow cytometry. Figure 5A shows that vemurafenib induced a dose-dependent G1 arrest. Furthermore, a significant fraction of the nuclei appeared in the sub-G1 phase, displaying weaker DNA-staining than expected for cells in G1. Following treatment with 1.25 µM vemurafenib, the number of cells found in G1 increased from 55% to 82%, while the number of cells in S and G2 phase decreased from 33% to 7.4% and from 11% to 2.3%, respectively, as quantified in Figure 5B and Table 2. Our results indicate that G1 arrest is the major contributing factor for the observed growth inhibition induced by vemurafenib in SA-4 cells.

### 2.5. Continuous Treatment with Vemurafenib Is Necessary to Sustain Growth Inhibition

**Figure 6:**
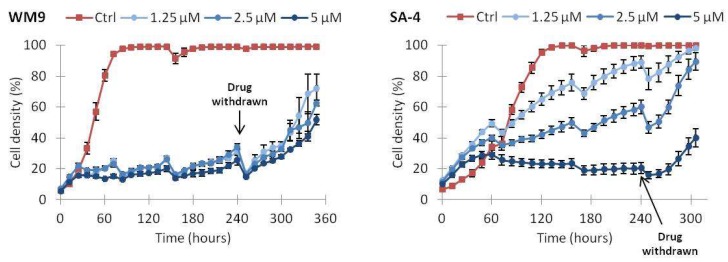
Continuous vemurafenib treatment is required for sustained growth inhibition. Cell lines were treated with the indicated concentrations of vemurafenib or vehicle only (Ctrl) for 240 h and growth medium supplemented with drug was replaced twice a week for this duration. As indicated by the arrows, after 240 h drug-containing medium was replaced with regular growth medium. Irregularities in growth curves are caused by loss of poorly attached cells by media change. One representative experiment is shown (*n* = 2). Error bars represent standard error of mean (SEM) of three technical replicates.

We also evaluated whether the effect of vemurafenib was transient or lasting after drug removal. SA-4 and WM9 cells were treated with 1.25, 2.5 or 5 µM vemurafenib for up to 10 days and subsequently incubated in growth medium without drug. As shown in Figure 6, again SA-4 required higher doses of vemurafenib for efficient growth inhibition, but even after the longest duration of treatment with the highest dose (5 µM), both SA-4 and WM9 cells regained growth after withdrawal of drug.

### 2.6. Vemurafenib De-Phosphorylates ERK Protein in All BRAF Mutated Sarcoma Cell Lines

**Figure 7:**
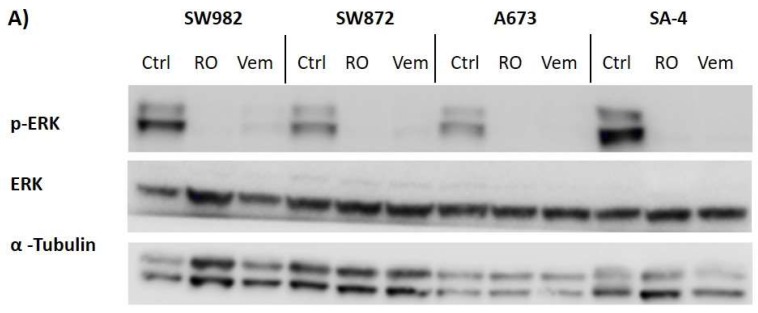

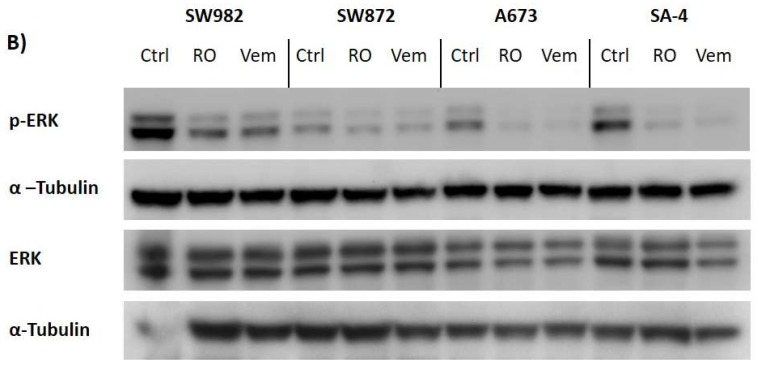
Vemurafenib inhibits ERK phosphorylation in sarcoma cell lines. Four BRAF^V600E^ mutated sarcoma cell lines were treated with 5 µM vemurafenib, 300 nM RO5126766 or vehicle only (Ctrl) for either (**A**) 3 h or (**B**) 72 h, before levels of phosphorylated ERK (p-ERK) in cell lysates were analyzed by western blotting, using indicated antibodies. α-Tubulin served as protein loading control.

ERK1 and −2 are the downstream kinases in the BRAF-MAPK signaling pathway, which phosphorylate nuclear transcription factors regulating cell growth and survival. Phosphorylation of ERK proteins can therefore be used as an indicator of an active MAPK signaling cascade. A common way for cells to circumvent BRAF inhibition is to up-regulate other signaling pathways that activate MAPK pathway, such as EGFR signaling, resulting in phosphorylation of ERK. To assess whether the sarcoma cell lines with a poor or short-term effect on cell growth circumvent the need for BRAF to be able to phosphorylate ERK, we evaluated the levels of p-ERK protein following treatment with vemurafenib for 3 or 72 h. The dual inhibitor RO5126766, targeting both BRAF and the downstream signaling molecule MEK, was included as a control. Treatment with either 5 µM vemurafenib or 300 nM RO5126766 for 3 h strongly reduced the level of phosphorylated ERK proteins in all the sarcoma cell lines harboring the mutation (Figure 7A). Levels of phosphorylated ERK were still strongly reduced after treatment for 72 h for all cell lines except for SW872 (Figure 7B).

## 3. Discussion

Precision cancer medicine is focused on targeting the tumor-specific mutations and affect mechanisms driving tumor growth. Repurposing drugs already approved for other indications are particularly valuable for orphan cancers such as sarcoma, where treatment options are limited and there is a large unmet therapeutic need. The V600E mutation of BRAF occurs in approximately 50% of melanoma tumors, and in these the selective BRAF^V600E^ inhibitor, vemurafenib, provides on average >7 months of progression-free survival, although as many as 50% of these patients do not respond in spite of having the mutation in their tumors [26]. BRAF^V600E^ is found in many cancer types, including some sarcomas [13,18,19,20,21,25,27], suggesting that vemurafenib might have activity against such tumors [21,28]. To determine the potential for using vemurafenib in sarcomas, we evaluated the efficacy in four BRAF^V600E^ mutated sarcoma cell lines, using a range of vemurafenib concentrations based on previous studies [21]. Vemurafenib has already been shown to have some activity in two of the cell lines included in our study, SA-4 and SW872 [21], but it was unclear how strong and lasting this effect was compared to a responding melanoma model, and also how this treatment affected signaling in such mesenchymal models.

The levels of mRNA and protein for both wild-type and mutated BRAF were similar in both SA-4 and SW872 sarcoma cell lines. However, the growth inhibitory responses to vemurafenib were variable, with a strong, dose-dependent effect observed for SA-4 and minor effects observed for SW872 and the remaining BRAF^V600E^ mutated sarcoma cell lines, A673 and SW982. In comparison, the melanoma cell line WM9 showed a strong and immediate growth inhibitory response also at lower doses, suggesting sub-optimal drug sensitivity in sarcomas. Acquired resistance to vemurafenib is commonly due to reactivation of MAPK signaling [29,30,31,32,33]. However, treatment with vemurafenib strongly reduced ERK phosphorylation in all the four mutated sarcoma lines at 3 h, and for three of the cell lines also at 72 h. A similar reduction was observed by treatment with the RAF/MEK-inhibitor RO5126766, indicating that SW982 and A673 cells are not intrinsically resistant to vemurafenib, but rather do not seem to be dependent on MAPK signaling for cell growth. However, ERK was fully phosphorylated again at 72 h in SW872 cells, which would explain the transient effect of vemurafenib on growth of these cells. Importantly, the presence of BRAF^V600E^ or de-phosphorylation of ERK upon vemurafenib treatment cannot be used to predict response in BRAF^V600E^ mutated sarcomas.

Further investigation of the growth inhibitory response observed for SA-4 cells revealed cell cycle arrest in G1 to be the major contributing mechanism for growth inhibition, consistent with previous studies in melanoma [34,35,36]. Although only moderate levels of apoptosis were induced in SA-4 cells, there was a long-lasting growth inhibitory effect at the highest drug concentrations. This could perhaps be a consequence of induction of senescence in some cells, as could be indicated by the weakly stained sub-G1 fraction observed by flow cytometry. However, continuous drug treatment was required to maintain the growth inhibitory response in both SA-4 sarcoma cells and the WM9 melanoma cell line, as cells regained growth following removal of vemurafenib at all tested concentrations. These findings indicate that there is a need for continuous treatment, should vemurafenib be used as a therapy against sarcoma, which is in agreement with experiences from treatment of other cancer types [37,38].

Our preclinical investigation of vemurafenib as treatment for patients with BRAF^V600E^ mutated sarcomas is limited by the lack of suitable models. The efficient response of SA-4 cells to vemurafenib appears to be an exception within sarcoma. This cell line was described by ATCC as a liposarcoma, but the subtype of liposarcoma is not known. The cells are able to differentiate into adipocyte-like cells with lipid droplets in vitro [39], and pathological assessment of HE stained sections of SA-4 xenograft tumors demonstrated the presence of cells harboring lipid droplets (Supplementary Figure S1), which supports the classification. The cells have a gain on chromosome 12, including CPM and MDM2 which is consistent with liposarcomas [40], but not highly amplified, which rules out a well-differentiated/de-differentiated liposarcoma subtype. The absence of the classical translocation (t12:16) indicative of myxoid liposarcomas [41] suggests that SA-4 is of the third subgroup of liposarcoma, the pleomorphic subtype, which lacks specific genetic aberrations used for diagnosis.

The BRAF^V600E^ mutation is only present in 2–5% of tumors derived from bone or soft tissue, according to the COSMIC database. Further, only 1 out of 4 BRAF^V600E^ mutated sarcoma cell lines in our study responded to treatment, demonstrating the need to better identify responders. Interestingly, clear cell sarcomas which have been shown to respond to vemurafenib treatment [19,25], often express melanoma-related markers. We found that SA-4 cells also express several genes typical of melanomas, such as MITF, MLANA, S100B, and TYR, which are otherwise poorly expressed in our panel of liposarcoma cell lines (Table 3). This might indicate that the presence of both mutated BRAF and melanoma markers could be used to identify sarcoma patients expected to respond to vemurafenib.

Although not yet supported by preclinical data, we would expect targeted treatment of BRAF^V600E^ mutated sarcomas to improve with the use of next generation RAF inhibitors in combination with other treatments. It could be envisioned that inhibition of phosphorylated ERK could sensitize other therapeutics or avoid resistance, and these options should be further evaluated.

## 4. Materials and Methods

### 4.1. Cancer Cell Lines and Cell Culturing

SA-4 (CRL-7938), SW872 (HTB-92), A673 (CRL-1598) and SW982 (HTB-93) cells were used as adherent human cancer cell model systems and were all obtained from ATCC. SA-4 is derived from a liposarcoma, whereas SW872 is derived from an undifferentiated liposarcoma. The A673 cell line represents Ewing sarcoma [42] and SW982 was derived from a synovial sarcoma, but lack the classical SYT-SSX translocation and is referred to as atypical synovial sarcoma [43]. The WM9 cell line (derived from a metastatic melanoma by Wistar Institute) and LPS510 cell line (derived from a dedifferentiated liposarcoma by Dr. J. Fletcher, Harvard Medical School, Boston, USA) were included as control cell lines in the study. All cell lines were cultured in a complete growth media consisting of RPMI1640 growth media (Sigma-Aldrich, St. Louis, MO, USA, #R0883) supplemented with 10% fetal bovine serum (FBS) (Sigma-Aldrich, #F7524), 1% L-alanine-L-glutamine (Glutamax) (Gibco by Life Technologies, Paisley, Scotland #35050-038) and 1% penicillin G and streptomycin sulfate (Sigma-Aldrich, #P4458). Cells were passaged twice a week and cultured in a humidified incubator holding 37 °C and 5% CO_2_. All cell lines included in the study were fingerprinted by short tandem repeat (STR) profiling to ensure correct identity of the cell stock and confirmed negative for mycoplasma infection using the VenorGeM Mycoplasma detection kit (Minerva Biolabs) for PCR.

### 4.2. Evaluation of Mutational Status in Cell Lines

Three of the sarcoma cell lines (SA-4, SW872 and LPS510) were exome-sequenced at the Oslo University Hospital Genomics Core Facility using Agilent SureSelect^XT^ Human All Exon v5 protocol and Illumina sequencing by synthesis technology on a HiSeq 4000 instrument (2 × 150 bp). Preprocessing, mapping of the reads and calling of variants was performed as previously described [44]. RNA-sequencing was performed at the Genomics Core Facility for three of the cell lines, SA-4, SW872 and LPS510, using the Illumina TruSeq Stranded mRNA Library Prep kit for NeoPrep following supplier’s instructions. The libraries were sequenced on a NextSeq 500 Illumina sequencer using a High Output v2 kit chemistry, generating 2 × 75 bp paired-end sequence reads. RNA-Seq reads were aligned using STAR aligner (v.2.5.0b) against the human reference genome (UCSC hg19, RefSeq and Gencode gene annotations), and FPKM estimation for each mRNA was generated by Cufflinks 2 using RNA-seq alignment app at Illumina BaseSpace. The RNA sequencing data for SW872 and SA-4 are available in the Short Read Archive (SRA), with accession number SRP 134156.

### 4.3. Western Blotting

Cells were resuspended in Lysis buffer (1% SDS, 0.3% Trizma Base, 7% glycerol and 3.5% β-mercaptoethanol, supplemented with phosphostop (#04906837001) and protease inhibitors (#04693124001), both from Roche, and subsequently boiled at 95 °C. Cell lysates were separated on a 4–12% PAGE gel (Novex by Life technologies, #NP0323BOX) with 3-(*N*-morpholino) propanesulfonic acid (MOPS) running buffer (Invitrogen, Carlsbad, CA, USA, #NP000102) and transferred to PVDF membrane (Merck, Kenilworth, NJ, USA, #IPVH00010). Membranes were blocked with TBS-T containing 5% bovine serum albumin (pERK) or 10% non-fat dry milk for the other antibodies and incubated over night while shaking. The antibodies used were as follows: pan-BRAF (1:200) (Sigma-Aldrich, #HPA001238), BRAF^V600E^ (1:1000) (RevMAb Biosciences, South San Francisco, CA, USA, #31-1042-005), phosphorylated p44/42 MAP kinase (pERK) (1:1000) (Cell signaling, #5370), p44/42 MAP kinase (ERK) (1:1000) (Cell Signaling, Danvers, MA, USA, #9102) and anti-α-Tubulin (1:2000) (Merck, #Cp06). Anti-Mouse (1:5000) (DAKO, #P0260) or anti-Rabbit (1:5000) (DAKO, Glostrup, Denmark, #P0448) was used as secondary antibodies. Following incubation with secondary antibody, the membrane was incubated in a development solution (Super Signal West Dura Extended Duration Substrate) (Thermo Scientific, Waltham, MA, USA, #34076). The emitted signal was detected using the digital developer G:BOX (Syngene) Cambridge, UK, and images captured were analyzed using the GeneSnap software.

### 4.4. Drug Treatment

The BRAF inhibitor, PLX4032 (vemurafenib) (Selleckchem, Munich, Germany #S1267) was dissolved in dimethyl sulfoxide (DMSO) to a 10 mM stock solution. The dual BRAF/MEK inhibitor RO5126766 (Selleckchem, #S7170) was dissolved in DMSO to a 1 mM stock solution. When used in experiments, drugs were further diluted in complete growth media. Cells treated with DMSO at a concentration corresponding to the highest drug concentration were included as controls in all experiments and referred to as vehicle.

### 4.5. Cell Growth by MTS

1000–2000 cells were plated in 96-well plates one day before drug treatment and subsequently treated with vemurafenib (0.31–5 µM) or vehicle only. Cell growth was assessed by MTS (Promega, Madison, WI, USA, #G5421) assay 72–96 h post treatment (depending on the growth rate of the individual cell lines), following the protocol recommended by the manufacturer. The absorbance was measured at 450 nm. The data was shown as mean of three biological experiments (*n* = 3) and presented relative to control.

### 4.6. Cell Cycle Analysis

10,000 cells were plated in 6-well plates, and on the subsequent day treated with the indicated concentrations of vemurafenib or vehicle only for 48 h. Cells were trypsinized, washed with PBS and fixed and permeabilized with 96% ethanol and stored at −20 °C at least 2 h. Cells were washed with PBS twice and stained with 2 µg/mL of the DNA-binding dye, Hoechst 33258 (Sigma-Aldrich, #94403). Approximately 1 × 10^6^ cells per sample was analyzed by flow cytometry using the LSR II (BD) at excitation/emission = 352/461 nm. FLOWJO v7.6.5 software and Watson settings were used to analyze the data. Data are presented as mean of three biological experiments.

### 4.7. Apoptotic Cell Death

1000–2000 cells were plated in 96-well plates and on the subsequent day, treated with vemurafenib at the indicated concentrations or vehicle only. The Cellplayer reagent that reports caspase 3/7 activity (Essen BioScience, Hertfordshire, UK #4440) was also included in a final concentration of 2.5 µM. The cells were monitored by live cell imaging (IncuCyte Zoom from Essen BioScience), where phase-contrast and green fluorescent images (488 nm) were recorded and used to estimate the confluence and the number of apoptotic cells, respectively. Data is presented as number of caspase-3/7 active cells per well and as means of three technical replicates. At 72 h, data is also shown as relative to cell density, normalized to control.

### 4.8. Growth Assay

1000–2000 cells were plated in 96-well plates one day before drug treatment, and subsequently treated with vemurafenib (1.25, 2.5 or 5 µM) or vehicle only. Replacement of media containing drug or control was performed twice a week for the entire duration of the experiment (approximately 240 h). To determine if continuous presence of drugs is necessary to achieve cell growth inhibition, drug containing media was exchanged with regular growth media after approximately 240 h. Cells were monitored by time-lapse microscopy, (IncuCyte FLR and IncuCyte ZOOM, Essen Bioscience) with phase-contrast images taken every third hour for the duration of the treatment, and cell growth estimated based on cell density. Data is presented as mean of three technical replicates.

### 4.9. Statistical Analysis

Data presented is obtained from at least three independent experiments, unless otherwise stated. Two-tailed paired Student’s *t*-test was used to compare the read-out of drug-treated with control-treated cells. *p* < 0.05 were considered statistically significant.

## 5. Conclusions

We conclude that vemurafenib can have therapeutic potential for selected cases of BRAF^V600E^ sarcoma; however others could benefit from vemurafenib in combination with additional therapies and further work is required to explore this.

## Figures and Tables

**Table 1:** mRNA expression.

GENE SYMBOL	SA-4	SW872	LPS510
ARAF	9.0	24.1	21.7
BRAF^WT^	4.3	4.4	2.7
BRAF^V600E^	4.0	4.6	0
RAF1	19.0	26.5	33.3

Gene expression shown as RPKM values (Reads Per Kilobase Million) based on mRNA sequencing data. RNA sequencing data for SW982 and A673 were not available.

**Table 2:** Percentages (%) of cells in the phases of cell cycle, including standard deviations (*n* = 3).

	G0/G1	S	G2	Sub-G1
Vehicle (Ctrl)	55.2 ± 4.8	33.1 ± 4.9	11.3 ± 1.7	0.5 ± 0.7
0.31 μM	72.4 ± 2.1	18.5 ± 3.8	5.5 ± 1.0	3.7 ± 2.7
1.25 μM	81.6 ± 0.7	7.4 ± 2.0	2.3 ± 0.8	8.7 ± 2.4

**Table 3:** Endogenous mRNA expression.

GENE SYMBOL	SA-4	SW872	LPS510
S100B	662	0.12	0
MITF	39.2	2.7	0.99
TYR	71.7	0.02	0
MLANA	278	0	0
SOX10	262	0.05	0

Gene expression shown as RPKM values (Reads Per Kilobase Million) based on mRNA sequencing data. RNA sequencing data for SW982 and A673 were not available and therefore not included.

## Supplementary Materials

The following are available online at http://www.mdpi.com/1422-0067/19/4/969/s1.

## Abbreviations

µ	Micro
µg	Microgram
µL	Microliter
µM	Micro molar
ARAF	v-Raf murine sarcoma 3611 viral oncogene homolog 1
ATCC	American type culture collection
BRAF	v-Raf murine sarcoma viral oncogene homolog B
BRAFV600E	V600E-mutated BRAF
BRAFWT	Wild-type BRAF
DMSO	Dimethyl sulfoxide
DNA	Deoxyribonucleic acid
EGF	Epidermal growth factor
EGFR	Epidermal growth factor receptor
ERK	Extracellular signal-regulated kinase
FBS	Fetal bovine serum
FDA	Food and drug administration
FPKM	Fragments per kilobase million
GIST	Gastrointestinal stromal tumor
MAPK	Mitogen-activated protein kinase
MEK	Mitogen activated protein kinase
MITF	Microphthalmia-associated transcription factor
mL	Milliliter
MLANA	Melanoma antigen recognized by T-cells 1
mM	Milli molar
mRNA	Messenger RNA
*n*	Number of biological replicates
nm	Nanometer
nM	Nano molar
*p*	Probability value
PAGE	Polyacrylamide gel electrophoresis
PBS	Phosphate-buffered saline
PCR	Polymerase chain reaction
PDGF	Platelet-derived growth factor
p-ERK	Phosphorylated ERK
PVDF	Polyvinylidene difluoride
RAF	Rapidly accelerated fibrosarcoma
RAF1	v-Raf-1 murine leukemia viral oncogene homolog 1
RAS	Ras sarcoma
RNA	Ribonucleic acid
RPKM	Reads per kilobase million
RT	Room temperature
RTK	Receptor tyrosine kinase
SD	Standard deviation
SDS	Sodium dodecyl sulfate
SEM	Standard error of mean
STR	Short tandem repeat
STS	Soft tissue sarcoma
TBS	Tris-buffered saline
α	Alpha
β	Beta
