# Supplementary material for: Preclinical Evaluation of Vemurafenib as Therapy for BRAFV600E Mutated Sarcomas
Source: Int J Mol Sci. 2018 Mar 23;19(4):969. doi: 10.3390/ijms19040969 (PMC5979358; doi:10.3390/ijms19040969)
Supplement: Supplementary file 1 [file ijms-19-00969-s001.pdf]

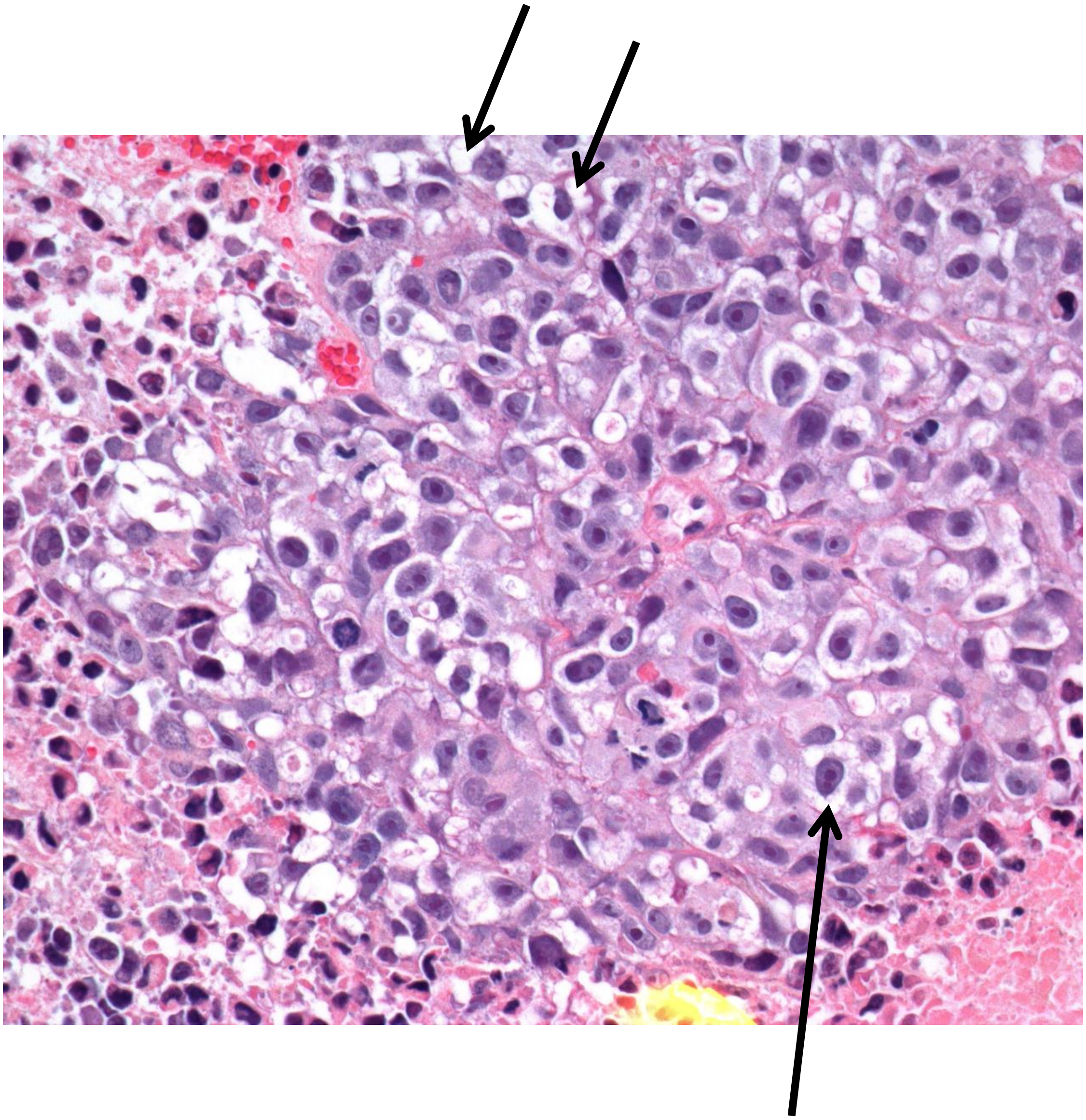

Supplementary Fig. S1: HE-stained section of SA-4 tumors grown in immunocompromised mice.
Arrows indicate cells with lipid droplets
